# Real-time nuclear magnetic resonance spectroscopy in the study of biomolecular kinetics and dynamics

**DOI:** 10.5194/mr-2-291-2021

**Published:** 2021-05-11

**Authors:** György Pintér, Katharina F. Hohmann, J. Tassilo Grün, Julia Wirmer-Bartoschek, Clemens Glaubitz, Boris Fürtig, Harald Schwalbe

**Affiliations:** 1 Institute for Organic Chemistry and Chemical Biology, Center for Biomolecular Magnetic Resonance (BMRZ), Johann Wolfgang Goethe-Universität Frankfurt, Frankfurt 60438, Germany; 2 Institute for Biophysical Chemistry, Center for Biomolecular Magnetic Resonance (BMRZ), Johann Wolfgang Goethe-Universität Frankfurt, Frankfurt 60438, Germany

## Abstract

The review describes the application of nuclear magnetic resonance (NMR) spectroscopy to study kinetics of folding, refolding and aggregation of proteins, RNA and DNA. Time-resolved NMR experiments can be conducted in a reversible or an irreversible manner. In particular, irreversible folding experiments pose large requirements for (i) signal-to-noise due to the time limitations and (ii) synchronising of the refolding steps. Thus, this contribution discusses the application of methods for signal-to-noise increases, including dynamic nuclear polarisation, hyperpolarisation and photo-CIDNP for the study of time-resolved NMR studies. Further, methods are reviewed ranging from pressure and temperature jump, light induction to rapid mixing to induce rapidly non-equilibrium conditions required to initiate folding.

## 1 Introduction

In 1993, the journal *Current Opinion in Structural Biology* published a special edition on protein–nucleic acid interactions edited by Dino Moras and Simon Philips and on protein folding edited by Chris Dobson. The edition featured an editorial article by Phillips and Moras (1993) on protein–nucleic acid interactions and reviews on transcription factor structure and DNA binding by Wolberger (1993), zinc-finger proteins by Berg (1993), DNA repair enzymes by Morikawa (1993), restriction endonucleases and modification methylases by Anderson (1993), DNA- and RNA-dependent DNA polymerases by Steitz (1993), aminoacyl-tRNA synthetases by Cusack (1993), work on ribosomes by Yonath and Franceschi (1993) and contributions by Robert (Rob) Kaptein on “protein-nucleic acid interaction by NMR” (Kaptein, 1993). This first part on protein–nucleic acid complexes was accompanied by a second part, introduced in the editorial article by Chris Dobson (1993), on protein folding with contributions by Dyson and Wright (1993) on peptide conformation and protein folding, on denatured states of proteins by Shortle (1993), on principles of protein stability by Fersht and Serrano (1993), on H/D exchange experiments by Baldwin (1993), on molecular simulation of peptide and protein folding by Brooks (1993), on protein folding by Dill (1993), on accessory protein in protein folding by Jaenicke (1993), and on antibody–antigen interaction by Wilson and Stanfield (1993).

Being invited to contribute to this edition of *Current Opinion in Structural Biology* nicely documents the eminent role that Rob Kaptein played as a nuclear magnetic resonance (NMR) spectroscopist in structural biology early on, in the heroic age of biomolecular NMR spectroscopy. Beyond the NMR community, his work was highly influential in the broad field of structural biology and known in this broad community. His main research focus, pursued together with Rolf Boelens in Groningen and in Utrecht, is the development of NMR spectroscopy for the determination of structure and dynamics of biomacromolecules, in particular for protein–DNA complexes. He was among the first to solve the structure of a sizeable protein, and he was among the first to push NMR towards 3D spectroscopy. Rob Kaptein is thus an NMR pioneer in bio-NMR. In addition to pushing the capabilities of NMR structure determination, the use of photo-CIDNP is intimately linked to Rob Kaptein.

The fundamental discovery of CIDNP goes back to Joachim Bargon and Hanns Fisher (Bargon et al., 1967) (see Bargon, 2006, and citations therein) and, independently, by Ward and Lawler (1967). Peter Hore provided key contributions (Hore and Broadhurst, 1993), but it was the work of Kaptein (Berliner and Kaptein, 1980; Buck et al., 1980; Kemmink et al., 1986a; Redfield et al., 1985; Scheek et al., 1979) that brought about the application of photo-CIDNP to biomolecular NMR spectroscopy. The explanation via the radical-pair mechanism and its description via the Kaptein rules are one prime example of his seminal contributions in this field (see Kaptein, 1975). They were independently confirmed by Closs and Closs (1969a, b). The CIDNP studies on flavins (Kaptein and Oosterhoff, 1969) initiated the application of photo-CIDNP to proteins (Kaptein et al., 1978) and brought about its broad use in biomolecular NMR spectroscopy (Hore and Kaptein, 1982), with numerous examples (Berliner and Kaptein, 1980; Buck et al., 1980; Kemmink et al., 1986a; Redfield et al., 1985; Scheek et al., 1979).

Kaptein showed the general applicability of photo-CIDNP NMR in probing the accessibility of aromatic amino acids in proteins to dyes and the concurrent manifold enhancement of NMR signal intensity through photo-induced dynamic nuclear polarisation. To conduct these photo-CIDNP experiments, Kaptein coupled high-power laser irradiation within the NMR tube in the NMR magnet and integrated light illumination into the NMR experiments. In situ illumination of dyes leads to signal enhancement of the aromatic acceptor amino acids tryptophan, in particular, tyrosine, and histidine, but also nucleobases in RNA and DNA. The possibility to couple laser light into the NMR tube and excite dyes homogeneously in samples dissolved in NMR tubes paved the way to utilise endogenous chromophores in proteins that are rigidly attached to a protein, as trigger for light-induced changes in protein conformation. In Kaptein's lab, one culmination of such approach was the study of photo-active yellow protein, which yielded important information on light-activated states of proteins, not obtainable by other structural biology techniques.

The research of Kaptein and seminal work pioneered in his group provided prerequisites for research interests in the group of the authors in developing and applying time-resolved NMR to a number of different systems including protein, RNA and DNA folding, refolding and aggregation. This contribution will thus focus on this topic. Anecdotally, a number of things should be added here. The work in our group was greatly influenced by Rob Kaptein, but also during a postdoctoral stay of one of us in Oxford by Peter Hore. Peter Hore conducted his postdoc with Kaptein, made great contributions, and carried on the torch of photo-CIDNP at times when the research focus of Kaptein and Boelens shifted more into biomolecular NMR spectroscopy. Here, he shifted his focus to the development of protein structure determination (Kaptein et al., 1985) in parallel with Kurt Wüthrich and 3D NMR spectroscopy (Vuister et al., 1990) in parallel with Christian Griesinger and Richard Ernst. Heinz Rüterjans, who long held the position for NMR spectroscopy in biochemistry at Goethe University Frankfurt, joined Kaptein's lab in Groningen, then still in Münster, to use photo-CIDNP to study the interaction of the Lac headpiece and DNA, which became a new research field with excellent possibilities in the Netherlands. Jacques van Boom had just developed DNA synthesis in the organic chemistry department of Leiden University, where Rob had done his PhD thesis. Kaptein formed a team with the biochemist Ruud Scheek. Scheek established headpiece and DNA purification and carried out the first NMR experiments of DNA complexes and worked on DNA assignment. Erik Zuiderweg from the Hilbers group in Nijmegen was in the team. He assigned the NMR spectra of the Lac headpiece and restrained molecular dynamics simulation (at the time!). And, of course, Rolf Boelens pushed the limits of 2D NMR on complexes of Lac and DNA. These studies defined the size limitation of 2D NMR at the time. Heinz Rüterjans' project was thus intimately linked to the projects that formed the basis of the success of the group in Utrecht around Rob Kaptein and Rolf Boelens. It should also be mentioned that one of us conducted his first laser-induced folding reactions on calmodulin together with Till Kühn in Utrecht using the laser installations at the Utrecht European NMR Large Scale facility in 1999. This review will thus summarise approaches to time-resolved NMR spectroscopy and coupling of methods to increase signal-to-noise in NMR for such time-resolved experiments.

## 2 Techniques to trigger real-time NMR experiments

NMR spectroscopy is unique in studying the kinetics of reactions and conformational transitions, including biomolecular folding and refolding with atomic-site resolution, and ever since the dawn of NMR, such applications have been pursued. Biomolecular folding can be studied at equilibrium or under non-equilibrium conditions and the theory describing the peculiar appearance of Fourier-transformed NMR spectra recorded during fast irreversible non-equilibrium reactions was developed early on (Kühne et al., 1979). While equilibrium studies focus on characterisation of conformational transitions in the microsecond-to-millisecond timescale involving NOESY-type experiments (Evans et al., 1989), lineshape analysis (Evans et al., 1989; Huang and Oas, 1995) or relaxation dispersion (Korzhnev et al., 2004), non-equilibrium studies focus on slower biomolecular folding transitions. The induction of non-equilibrium conditions can be conducted in an irreversible manner. A prime example of the kinetic studies under irreversible conditions are experiments that utilise a rapid change in solution conditions, a so-called mixing step, and subsequent spectroscopic quantification of the build-up of a new equilibrium under the conditions after mixing. This is commonly accomplished by mixing-based NMR approaches comprising stopped flow (Frieden et al., 1993; McGee and Parkhurst, 1990), rapid injection (Balbach et al., 1995) as well as mixing-probe technologies (Spraul et al., 1997, and reviewed in Schlepckow et al., 2011) or laser-based experiments (Kühn and Schwalbe, 2000). The time resolution of the experiments is mainly determined by acquisition of sample homogeneity. Dead times can be as short as 50 ms nowadays (Mok et al., 2003; Schlepckow et al., 2008). A further extension of time-resolved NMR is to utilise freeze quenching as an approach that couples rapid induction of biomolecular folding with rapid freezing of the acquired conformational ensemble at appropriate time points after folding induction to study its properties by solid-state NMR spectroscopy (Etzkorn et al., 2007; Jeon et al., 2019).

A fundamentally different approach to studying biomolecular folding and refolding reactions is to change the state parameters pressure or temperature to rapidly induce non-equilibrium conditions. If the pressure or T jumps do not induce alterations in the macromolecular system of interest, in particular if temperature- or pressure-induced aggregation can be circumvented, then changes in state parameters are reversible and can thus be applied multiple times, allowing for sophisticated multidimensional NMR detection schemes. In the following Sect. 2, we discuss the available methods to conduct such time-resolved NMR experiments.

### 2.1 Rapid mixing

**Figure 1:**
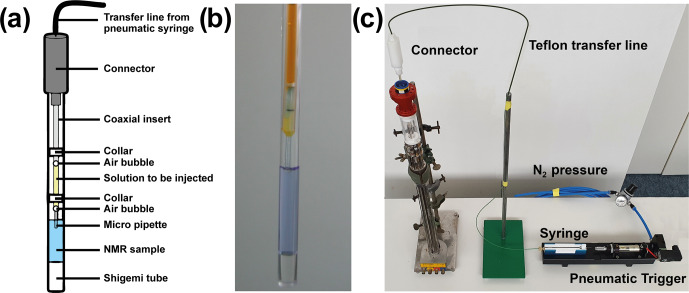
Setup to trigger real-time NMR experiments with in situ rapid mixing. **(a)** Schematic representation of the rapid-mixing device introduced by Mok et al. (2003). **(b)** Shigemi tube with injection insert. Air bubbles prevent premature mixing via a liquid–liquid interface. **(c)** Rapid-mixing setup used at BMRZ, Frankfurt. The sample is connected via a Teflon transfer line to an external syringe that induces rapid mixing after a pneumatic trigger.

The first real-time NMR investigations of protein folding induced by rapid mixing were conducted in 1988. Applications focused on coupling of protein folding and hydrogen exchange (Elove et al., 1994; Radford et al., 1992; Roder et al., 1988; Udgaonkar and Baldwin, 1988) and became particularly popular in the mid-90s (Balbach et al., 1996, 1995; Hoeltzli and Frieden, 1995; Kiefhaber et al., 1995; Van Nuland et al., 1998). Rapid-mixing experiments typically use a setup with a Teflon transfer line, filled with buffer solution that connects the NMR tube with an injection piston outside the magnet. The sample solution is loaded into the transfer line and separated from the solution in the NMR tube by an air bubble that precludes premature mixing at a liquid–liquid interface (see Fig. 1). A pneumatic trigger induces rapid injection (Van Nuland et al., 1998). This simple rapid-mixing setup has been revised to an in situ device ready to use in conventional NMR probes with a dead time after injection of 50 ms (Mok et al., 2003). More recently, a 3D-printed rapid-mixing device with optimised injection has been reported (Franco et al., 2017a). A removable injector allows use of smaller sample volumes and minimises the disturbance of the magnetic field homogeneity. While originally not intended for measuring kinetics, dissolution DNP (see below) also uses rapid mixing to inject polarised water into the NMR tube, allowing triggering kinetics by adding a folding cofactor simultaneously with the polarised water, as shown recently (Chen et al., 2013; Novakovic et al., 2020a).

Biomacromolecules can be unfolded in many different ways (Fürtig et al., 2007b; Roder et al., 2004). Proteins can be chemically denatured using high concentrations (6–8 M) of guanidinium chloride (GdnCl) (Logan et al., 1994; Zeeb and Balbach, 2004) or urea (Egan et al., 1993; Neri et al., 1992; Schwalbe et al., 1997), but also organic solvents including 2,2,2-triflouroethanol (TFE) or dimethyl sulfoxide (DMSO) (Buck, 1998; Buck et al., 1995, 1993; Nishimura et al., 2005). The (re-)folding of chemically denatured proteins can then be initiated with a rapid dilution into native buffer conditions (Balbach et al., 1995) or vice versa for unfolding of native proteins (Kiefhaber et al., 1995). Alternatively, a rapid pH change can be used to re- or de-nature proteins (Balbach et al., 1996; Corazza et al., 2010; Dobson and Hore, 1998; Schanda et al., 2007; Zeeb and Balbach, 2004). The rapid-mixing design introduced by Mok et al. (2003) has also found widespread application for studies on folding of nucleic acids (see below).

### 2.2 Pressure jump

Pressure is a physical state parameter that can influence the conformation of biomolecules. High pressure can alternate the stable conformation, such to reveal intermediate like conformations (Kitahara et al., 2005; Kitahara and Akasaka, 2003) as well as denature proteins. This process is usually reversible, and upon release of pressure the protein folds back to its native state. For more in-depth application and thermodynamic discussion about pressure-induced conformational changes, we refer to the following reviews (Akasaka, 2018, 2006; Lassalle and Akasaka, 2006). The required pressure can be adjusted by using chaotropic agents or temperature to lower the overall stability or by introducing specific mutations to introduce internal cavities in the folded structure (Bouvignies et al., 2011; Mulder et al., 2001). Static high-pressure NMR spectroscopy is a long-established method to assess the thermodynamic profile of proteins (Balbach et al., 2019) and allows detailed thermodynamic characterisation of the energy landscape (Akasaka et al., 2013; Roche et al., 2019). There are further reviews about equilibrium high-pressure measurements as reviewed in Roche et al. (2017), Caro and Wand (2018) and Nguyen and Roche (2017). Here, we focus on more recent developments regarding the rapid change in pressure inside the spectrometer to study kinetics of biomolecules, especially protein-folding kinetics.

**Figure 2:**
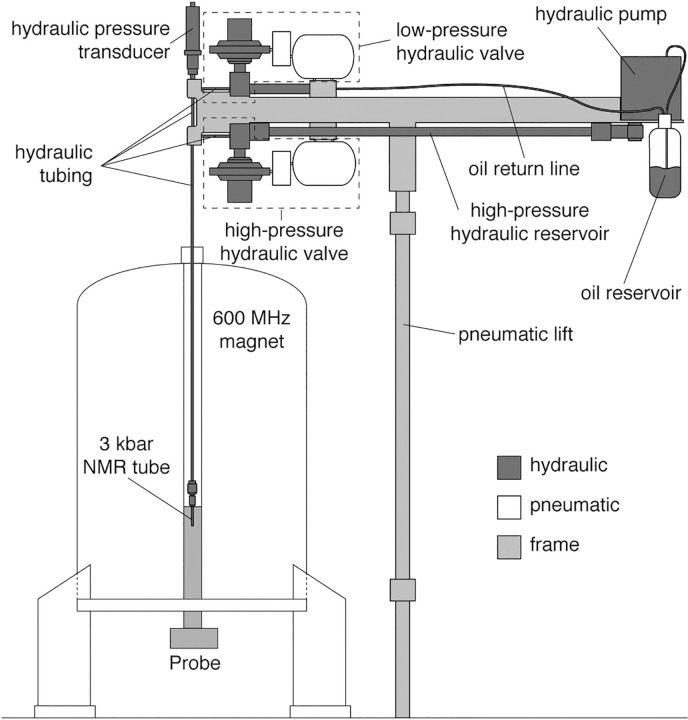
Schematic representation of the rapid pressure-jump NMR apparatus, developed by Charlier et al. (2018c). The apparatus is mounted onto a frame with pneumatic lift (light grey) to adjust the height to any spectrometer. The pressure apparatus is a closed system under N ${}_{2}$ atmosphere and uses a high-pressure and atmospheric-pressure reservoir to increase or to decrease the pressure correspondingly inside the sample stored in a Zirconia tube inside the spectrometer. Reprinted with permission from Charlier et al. (2018c).

High-pressure NMR measurements require a special NMR tube (made from either quartz, sapphire or zirconia) that can withstand high pressures of up to a few thousand bar. The most often used ones are made from zirconium oxide, as they are commercially available and are specified up to a maximum of 3000 bar (Daedalus Innovations LLC). The pressure is usually realised by use of an external hydrostatic pressure pump connected via pressure-withstanding tubing to the sample inside the spectrometer. Usually, mineral oils are used to transmit the pressure providing phase separation from the typical water samples. The currently most advanced setup was developed by Charlier et al. (2018c), and the schematic is shown in Fig. 2. The system uses a high-pressure and an atmospheric-pressure reservoir, connected through a hydraulic valve to the sample. By opening the valve from the high-pressure reservoir, the pressure is rapidly equilibrated in the sample to the high pressure, while the pressure rapidly drops in the sample by changing to the low-pressure reservoir. The oil reservoir is kept in a closed system under nitrogen atmosphere to avoid oxidation.

The major advantage of pressure jump compared to other methods to study protein folding and energy landscape lies in the reversibility of the induced conformational transition. In combination with the shortest time (1–5 ms) requirement to change between folding and unfolding conditions, this method allows complex NMR experiment designs to study in detail the folding pathways, mechanism and even the structure of intermediates.

### 2.3 Light induction

**Figure 3:**
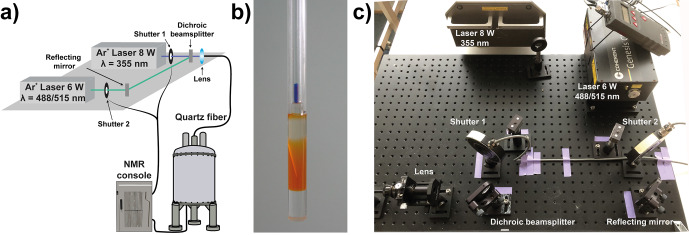
Setup to trigger real-time NMR experiments with in situ light induction. **(a)** Schematic representation of a two-source setup for high-power laser irradiation connected to a NMR tube via a quartz fibre. The shutters can be directly triggered via the NMR console/pulse programme. **(b)** Shigemi tube with cone-shaped quartz tip for homogeneous sample illumination (Kühn and Schwalbe, 2000). The glass fibre can be inserted into the plunger. **(c)** Two-wavelength laser setup installed at BMRZ, Frankfurt.

Folding can be initiated photochemically by irradiation within the NMR spectrometer. Laser irradiation of biomolecules within the NMR spectrometers was introduced by Kaptein in photo-CIDNP NMR (Kaptein et al., 1978), before the first real-time folding applications were conducted. In folding applications, high-power laser irradiation (up to 8–10 W primary output) is coupled to the NMR spectrometer by a quartz fibre ending in the NMR tube within the spectrometer. Figure 3 shows the setup of two lasers coupled to an NMR spectrometer as it is used at BMRZ in Frankfurt. To achieve reasonable irradiation times (typically between 0.2 and 4 s) depending on the folding rate to be observed, not only the applied power is important, but also the homogeneous light illumination within the sample. Different methods that advance the setup presented by Berliner (Scheffler et al., 1985), such as using a cone-shaped quartz tip (Kühn and Schwalbe, 2000) or stepwise-tapered (Kuprov and Hore, 2004) or sandblasted quartz fibre ends (Feldmeier et al., 2013), have been introduced to achieve this.

Most importantly, the approach relies on the presence of a chromophore within the NMR sample. This can either be a biomolecule carrying a photoactive group such as the yellow protein (Derix et al., 2003) or folding can be initiated by release of cofactors from photo-labile chelators (Kühn and Schwalbe, 2000), from caged ligands (Buck et al., 2007) or from photo-labile precursors that cage biomolecular conformation (Wenter et al., 2005).

### 2.4 Temperature jump

Next to pressure, temperature is the second thermodynamic state parameter. It is coupled to the enthalpy change in a conformational equilibrium. Thus, the change in temperature was one of the first methods to initiate changes in biomolecular systems, and ultrafast T -jump experiments were introduced by Manfred Eigen and awarded with the Nobel Prize in Chemistry in 1967. The first application of temperature jump in combination with NMR spectroscopy was the study of proline *cis-trans* isomerisation in oligopeptides as an alternative to the jump in pH(D) by Wüthrich (Grathwohl and Wüthrich, 1981). Additionally, temperature changes can initiate refolding of biomolecules exhibiting temperature-dependent conformations (Reining et al., 2013; Rinnenthal et al., 2010), denature proteins at high temperature, or refold cold-denatured proteins.

**Table 1:** Comparison of different T -jump initiation methods used in combination with NMR spectroscopy.

T -jump method		Heating	Homogeneity	Temperature	Special	Temperature	BioNMR application
		speed		range	requirement	stability	
Laser (Ernst et al., 1996;		Fast	Low	Excellent (up to	Laser setup	No	–
Ferguson et al., 1994)				few hundred K)			
Gas heating (Akasaka		Slow (in both	Good	Small	Vibration	Yes	Refolding of RNase A
et al., 1990)		direction)			stabilisation		from thermal-denatured
							state (Akasaka et al.,
							1991)
Flow system		Moderate	Good	Moderate	Stop-flow	Yes	Folding of heat-denatured
					system		RNase A (Yamasaki et
							al., 2013)
Dielectric	Microwave	Fast	Moderate (good with	Moderate–good	Special coil	No	RNase A denaturation
heating	( > 1 GHz)		mechanical mixing		design		(Naito et al., 1990)
			device) (Kawakami				
			and Akasaka, 1998)				
	Radio-	Moderate–fast	Good	Moderate	Special coil	In combination	Folding of cold-denatured
	frequency				design	with gas	barstar (Pintér and
	( < 1 GHz)					heating	Schwalbe, 2020;
							Rinnenthal et al., 2015)

Later, different technical setups were developed to speed up the temperature change to study dynamic changes with faster reaction rates. A list of the different techniques to achieve a temperature jump is given in Table 1. In this table, important parameters are described for each technique. Out of all the different T -jump techniques, microwave (MW) and radio-frequency (RF) heating proved to be the most suitable for biomolecular NMR. In both cases, inductive and dielectric heating effects take place; the latter is the major factor and couples well to lossy samples (salt-containing aqueous samples). RF heating allows easy coupling to the spectrometer, due to built-in RF generators and an amplifier system. It can reach relatively fast heating with 20 K s ${}^{-1}$ , although slower than MW setups, but offers a more homogeneous heating profile which is required for high-resolution NMR spectroscopy.

**Figure 4:**
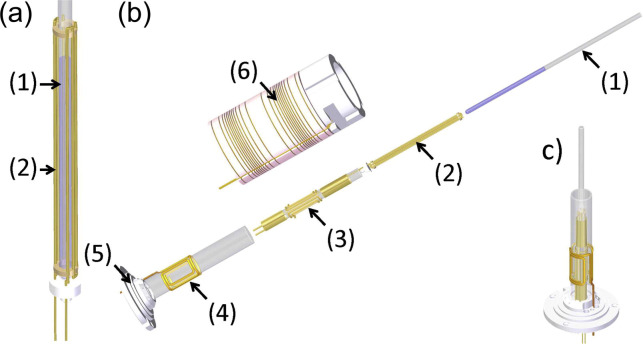
Schematic view of the coil assemblies in the T -jump probe head developed by Rinnenthal et al. (2015). **(a)** Cage coil/wire capacitor for rf heating with sample tube (2.5 mm), **(b)** exploded view of the overall assembly with (1) sample tube, (2) 2.5 mm cage coil, (3) 5 mm double-tuned ( ${}^{1}$ H and ${}^{2}$ H) rf NMR saddle coil, (4) 10 mm ( ${}^{15}$ N) rf NMR saddle coil, (5) coil insert base and connection to the main probe body, and (6) z gradient coil. **(c)** Full assembly of coils in the T -jump probe ( z gradient system not shown). Reproduced with permission from Rinnenthal et al. (2015).

The latest RF heating setup, shown in Fig. 4, described in the literature (Rinnenthal et al., 2015) uses a built-in RF coil to initiate the jump with an additional optimised gas heating to stabilise final temperature. The range of the temperature jump can be adjusted by the number of heating RF pulses applied, and for longer measurements, the gas heating provides stability at the final temperature. The suitability of this setup for studying the folding mechanism of proteins has been demonstrated on the cold-denatured barstar, where the temperature jump initiated the complete reversible refolding of the protein.

### 2.5 General pulse scheme for RT-NMR

**Figure 5:**
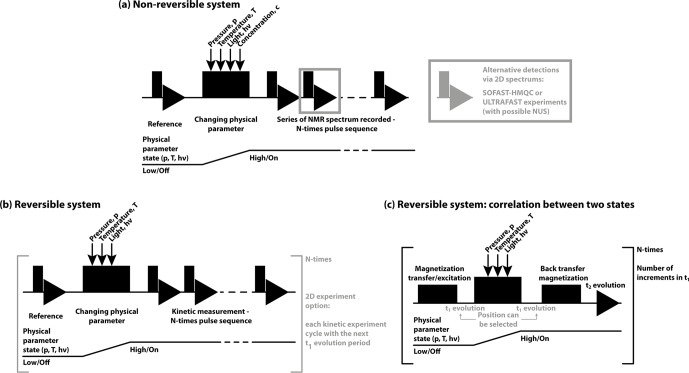
General pulse schemes used for RT-NMR measurements. In all cases the first reference spectrum is measured, followed by a rapid trigger using the corresponding physical parameter change. In **(a)** the general scheme is shown for non-reversible systems and in **(b)** the reversible systems are shown. In **(c)** the basic scheme to correlate the starting and final states (Naito et al., 1990) is shown, suitable for reversible systems to study rapid changes ( < 1 s).

In the context of real-time NMR, a number of aspects within pulse sequences have to be conceptualised. Firstly, the timing of NMR excitation, synchronous triggering of folding, and the correlation of NMR coherences or polarisations have to be designed (shown in Fig. 5). Secondly, the best excitation pulses and detection schemes have to be applied. The pulse sequences used to measure time-resolved NMR experiments depend on the trigger and on the system under study. These can be divided into two major groups: non-reversible (Fig. 5a) or reversible (Fig. 5b–c) systems. In both cases, before initiating the kinetic experiment, reference spectra are recorded. For non-reversible systems, the basic scheme is a simple trigger after which a series of 1D-NMR spectra is recorded, allowing the best time resolution. While 2D experiments can provide higher chemical shift resolution, they can only be utilised for slow kinetic measurements. Depending on the timescale of the observed kinetics, ${}^{15}$ N / ${}^{13}$ C– ${}^{1}$ H correlation spectra can be measured. Modifications to these experiments can speed up the recording and further increase the time resolution. These techniques include different SOFAST and BEST-HMQC techniques (Favier and Brutscher, 2011; Schanda et al., 2006, 2005; Schanda and Brutscher, 2005) with longitudinal relaxation optimisation (Farjon et al., 2009), Hadamard frequency encoding (Schanda and Brutscher, 2006) or ultrafast approaches (Gal et al., 2007) or, in the case of NOESY experiments, Looped-PROjected SpectroscopY (L-PROSY) (Novakovic et al., 2020b, 2018). Furthermore, several of the pulse sequences can be combined with non-uniform sampling (NUS) (Gołowicz et al., 2020) to further reduce the final measurement time up to a few seconds.

For reversible systems, 2D experiments can be recorded with the same time resolution as 1D-NMR experiments. To achieve this, only one increment of the indirect dimension is recorded in every kinetic experiment, and time incrementation in the indirect dimension is achieved after each new folding trigger. Repeating the experiments and measuring the required indirect dimension points and finally concatenating together the corresponding time points, kinetic measurements with high temporal and spectral resolution can be achieved (Naito et al., 1990; Harper et al., 2004; Kremer et al., 2011; Alderson et al., 2017).

It is noteworthy that reversible systems in combination with magnetisation transfer between starting state one (at the start) and state two (new equilibrium) allow recording of a so-called state-correlated (SC) spectrum (Kawakami and Akasaka, 1998) and its adopted versions (Rubinstenn et al., 1999; Charlier et al., 2018c, b; Pintér and Schwalbe, 2020). In the SC spectrum, the pulse sequence starts with magnetisation transfer to other nuclei before the actual physical parameter (light, pressure or temperature) is changed. The final detection takes place already in the new equilibrium state. The limiting factor for such application is the speed of the physical parameter change, as it has to be faster than the $T_{1}$ relaxation time. Additionally, double-jump experiments can also be utilised to observe alternative folding pathways if rapid changes between state one and state two in both directions are possible (Charlier et al., 2018a; Kremer et al., 2011; Pintér and Schwalbe, 2020).

## 3 Signal enhancement

It is beyond the scope of this article to cover the physical principles of the plethora of signal-to-noise enhancement approaches in NMR spectroscopy. Time-resolved experiments, however, provide very stringent requirements for signal-to-noise as the kinetics of structural transitions in biomolecular folding define the time window that is available. In the following, we will discuss the coupling of time-resolved experiments to dynamic nuclear polarisation (DNP) in solid-state NMR (Becerra et al., 1993; Corzilius, 2020), to hyperpolarisation (Ardenkjær-Larsen et al., 2003; Ragavan et al., 2011) and to photo-CIDNP experiments in liquid-state NMR.

### 3.1 DNP

Dynamic nuclear polarisation describes a set of mechanisms by which nuclear non-Boltzmann magnetisation is created (for a recent review, see Corzilius, 2020). So far, it works best for solid-state NMR, and especially polarisation transfer via the cross-effect using biradicals as polarisers has progressed from the proof-of-concept stage to numerous applications. Enhancements of up to 150-fold have been reported on complex systems such as GPCRs (Joedicke et al., 2018) or ribosome nascent chain complexes (Schulte et al., 2020). This significant boost in sensitivity can be highly beneficial for real-time NMR applications. However, a sufficiently long electron relaxation for an efficient polarisation transfer to the nuclei is required, which has to be obtained under cryogenic conditions. Therefore, DNP-enhanced solid-state NMR experiments are usually performed around 100 K or even below, making them less compatible with real-time studies but offering great opportunities for cryotrapping of intermediate protein states. The first attempts, initially without the help of DNP, date back to 1994 (Ramilo et al., 1994). Recently, remarkable progress has been achieved by combining rapid mixing and freeze quenching with DNP by which a time resolution in the millisecond range could be achieved (Jeon et al., 2019). Another possibility is cryotrapping of light-induced photoreceptor intermediates under DNP conditions, which is briefly addressed below.

### 3.2 Dissolution DNP – hyperpolarisation

The application of DNP enhancement schemes employing direct microwave irradiation and use of polarising agents is limited in liquid-state NMR. Due to the large dielectric losses in water, the sample volume is usually limited to the nanolitre range. However, polarisation of the solvent in the solid state followed by rapid heating and injection e.g. of polarised solvent can be utilised for signal enhancement in real-time NMR measurements of biomolecules. It was first demonstrated by Ardenkjær-Larsen (Ardenkjær-Larsen et al., 2003) and used for example to enhance the sensitivity of ${}^{13}$ C-detected in vivo metabolic MRI (Kurhanewicz et al., 2019) and later developed by the Frydman group for biomolecules.

The solvent water, in a pellet form, is hyperpolarised at low temperature in a hyperpolariser. The pellet is heated to liquid state by flushing with hot solvent. The commercially available Hypersense (Oxford Instruments Plc) instrument uses 3–4 mL of hot water, while the Frydman group has developed an elegant method to reduce the dilution factor that also extracts the radical agent, using hot non-polar solvents (Harris et al., 2011). The latter has the advantage of removing the polarising agent which would cause line broadening. The polarised solvent is then injected ( ∼ 2 s) into the sample already placed inside the spectrometer.

This method has been demonstrated on the intrinsically disordered protein p27 ${}^{\mathrm{Kip}1}$ , which refolds upon interaction with Cdk2/cyclin (Ragavan et al., 2017). Direct polarisation of p27 ${}^{\mathrm{Kip}1}$ in deuterated form was used to increase $T_{1}$ relaxation time.

The group of Hilty was the first to demonstrate the applicability of dissolution DNP to folding experiments (Chen et al., 2013). In this application, the ribosomal protein L23 was hyperpolarised and subsequently injected to a folding buffer of higher pH, and folding is monitored by ${}^{13}$ C 1D spectra.

In a more recent application, Novakovic et al. (2020a) used dissolution-DNP to monitor the real-time refolding of the RNA aptamer domain of a guanine-sensing riboswitch (GSW) upon ligand binding (Novakovic et al., 2020a). Different to proteins, the exchange of solvent water with imino sites in RNAs is sufficiently fast not only in unstructured regions, but also in structured regions of the RNA, and thus all RNA sites can benefit from increased signal-to-noise due to exchange transfer. In this paper, the GSW-specific ligand (hypoxanthine) was injected parallel with the hyperpolarised solvent water into the RNA sample. Co-injection of hyperpolarised water and refolding initiating ligand induced refolding of the aptamer, observable with an almost 300-fold imino signal enhancement. The obtained signal enhancement is sufficient to even record series of the 2D-NMR spectrum using a selective HMQC pulse sequence.

### 3.3 Photo-CIDNP

Besides microwave-driven DNP, chemically induced dynamic nuclear polarisation (CIDNP) is a method that can selectively enhance the signal-to-noise of NMR signals. Especially photo-CIDNP offers the possibility of probing the solvent accessibility of amino acids in proteins and peptides. During protein folding and refolding, the solvent-accessible area of a protein changes due to hydrophobic collapse that rearranges the conformation of solvent-exposed amino acids. This probing of differential accessibility makes photo-CIDNP a powerful tool for the (real-time) investigation of protein folding (Hore and Broadhurst, 1993; Kuhn, 2013; Morozova and Ivanov, 2019).

The positive or negative signal enhancement is based on a chemical reaction between a laser-induced excited photosensitiser and a CIDNP-active aromatic group. The radical pair mechanism explains the photo-CIDNP effect with an excited photosensitiser that is present in the triplet state, after intersystem crossing, accepting an electron or hydrogen from a donor molecule. A radical ion pair is formed and the singlet recombination probability of this pair is influenced by the hyperfine coupling constants (Hore and Broadhurst, 1993). The hyperfine coupling leads to differences in the population of the nuclear spin energy levels and therefore emissive or absorptive NMR signals, predictable by Kaptein's rule (Kaptein, 1971). The setting of a basic photo-CIDNP pulse sequence is quite uncomplicated. After an optional presaturation, the sample is illuminated for a short time controlled by a mechanical shutter followed by the desired experimental pulse sequence. Alternating light (with laser irradiation) and dark (without irradiation) spectra are recorded and subtracted to obtain the difference spectra with the enhanced signals (Hore and Broadhurst, 1993). Several amino acids exhibit polarisation by photo-CIDNP in solution: these are tryptophan, tyrosine, histidine and also methionine, glycine and methylcysteine, although to different extents (Stob and Kaptein, 1989; Morozova et al., 2005; Morozova and Yurkovskaya, 2008; Morozova et al., 2016). Embedded in a protein or peptide, Trp, Tyr, His and Met show signal enhancement if they are accessible to a photosensitiser (Kaptein et al., 1978; Hore and Broadhurst, 1993). For the photo-CIDNP reaction different dyes as photosensitiser can be used, the most common ones are substituted flavins, 2,2-dipyridyl (DP), 4-carboxy-benzophenone (4-CBP), and 3,3',4,4'-tetracarboxybenzophenone (TCBP) (Morozova and Ivanov, 2019).

The first presented photo-CIDNP studies on proteins were published by Kaptein et al. (1978) on bovine pancreatic trypsin inhibitor (BPTI). The investigation of BPTI by photo-CIDNP showed that the enhanced signals of tyrosine residues were in line with ones exposed on the surface in the crystal structure. In the following years, the time-resolved investigation of the kinetics of folding or refolding proteins with photo-CIDNP should become more and more important. This is also due to the fact that time-resolved photo-CIDNP studies have a better time resolution than other NMR techniques, because of the laser-induced generation of nuclear polarisation. The repetition rate is determined on electron relaxation, not nuclear relaxation rates (Day et al., 2009; Kuhn, 2013). Also, the small chemical shift resolution in non-native or not folded states of proteins can be overcome due to the fact that only solvent-accessible amino acids are photo-CIDNP-sensitive, and the investigation of not folded or partially folded structures at a residue-specific level is possible (Schlörb et al., 2006). During folding, several conformational states, including random coil, molten globule states as well as folding intermediates, non-native states, partially folded states and native states can be characterised in a residue-specific manner (Kuhn, 2013).

Besides amino acids, DNA and RNA mononucleotides are also CIDNP-active, including guanosine, adenosine and thymidine (Kaptein et al., 1979; Pouwels et al., 1994). With a self-complementary tetramer it was shown that photo-CIDNP can only be detected in single-stranded regions, when the nucleobase is accessible to the solvent and the photosensitiser (McCord et al., 1984a). Photo-CIDNP studies on tRNA were the first investigations of such a kind on larger nucleic acids. Temperature-dependent photo-CIDNP experiments showed changes consistent with the melting of tertiary and secondary structures (McCord et al., 1984b). Therefore, photo-CIDNP can also be a powerful tool for the characterisation of the accessibility of nucleobases in RNA or DNA that play key roles in RNA–protein binding sites.

## 4 Overview of light and rapid-mixing applications

Folding induction using rapid-mixing approaches coupled to NMR is universally applicable and the installation of the rapid-mixing apparatus is not costly. Experimental challenges, including deterioration of NMR spectra, remain, however. The coupling of laser irradiation to trigger biomacromolecular folding reactions is conceptually more elegant but places more stringent requirements on the systems under study. Advantages are obvious: the dead time of folding induction is no longer determined by build-up of NMR homogeneity and the absence of flow-related susceptibility inhomogeneities across the sample but homogeneous illumination that depends on photophysical properties, including concentration-dependent extinction coefficients. Triggering times can thus be shortened as signal-to-noise is increased. A number of biomacromolecules carry an endogenous chromophore and selected examples of NMR spectroscopic investigations of these systems are discussed in Sect. 4.1.

Other systems can be modified by photo-active non-natural groups or, alternatively, cofactors or ligands can be masked by a photolabile group. These applications are discussed in Sect. 4.2 along with applications of rapid mixing as the two methods complement each other in some of the systems investigated.

### 4.1 Endogenous chromophores

#### 4.1.1 Photo-active yellow protein

**Figure 6:**
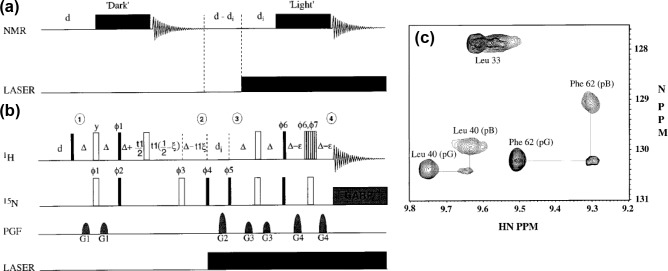
General NMR scheme for the study of pB **(a)** and ${}^{15}$ N-SCOTCH subsequence for HSQC assignment **(b)**. **(c)** Superimposed regions of the HSQC spectra of pG, pB, and the ${}^{15}$ N-SCOTCH exchange experiment connecting the HSQC cross peaks. Reproduced with permission from Elsevier (Rubinstenn et al., 1999).

The photo-active yellow protein (PYP) from the Ectothiorhodospira halophile shows negative phototactic response towards intense blue light (Sprenger et al., 1993). Light excitation of PYP induces a photocycle during which PYP undergoes structural and dynamic changes. The photocycle is defined by three states, the ground state pG, the red-shifted intermediate pR and the long-lived blue-shifted intermediate pB. The photocycle is coupled to the formation of a covalently bound thiol ester-linked chromophoric group, the p-coumarin acid, which undergoes *cis-trans* isomerisation upon light excitation (Hoff et al., 1994). Early on, Kaptein realised that due to the small size of the protein (14-kDa) and the chromophore, PYP is an excellent model system for the investigation of the processes occurring during photoreception in solution at atomic resolution. Kaptein's group thus investigated the blue-shifted intermediate pB of the photocycle of PYP (Düx et al., 1998; Rubinstenn et al., 1999, 1998). By combination of light induction and NMR, the structure and backbone dynamics of the long-lived intermediate pB and the ground-state pG were characterised. Irradiation with argon light inside the NMR tube generated the kinetic intermediate, while its properties were studied by multidimensional heteronuclear experiments. Besides conventional correlation and relaxation experiments, Rubinstenn et al. (1999) developed a 2D ${}^{1}$ H– ${}^{15}$ N-SCOTCH (Spin Coherence Transfer in Chemical Reactions) (Rubinstenn et al., 1999) experiment as a further development of the previously introduced ${}^{1}$ H NMR SCOTCH experiment (Kemmink et al., 1986b). With this, the long-lived intermediate pB populated on the photocycle could be generated by light, and its resonances could be correlated with the pG state as an early example of a state-correlated 2D NMR experiment. In the SCOTCH experiment, the ${}^{15}$ N chemical shift of pG is correlated with the ${}^{1}$ H chemical shift of the ${}^{15}$ N-attached proton of pB (Fig. 6b, c). The experiments revealed that pB is structurally disordered in solution populating an ensemble of conformers in exchange on a millisecond timescale. This finding was in contrast to previous crystal structures that showed a single structure for state pB with just minor changes around the chromophore (Genick et al., 1997). Further investigations of PYP in solution with hydrogen–deuterium exchange, pH studies, a mutant lacking negative charge and an N-terminally truncated variant revealed more detailed information about the protein and its photo-active cofactor (Bernard et al., 2005; Craven et al., 2000; Derix et al., 2003).

#### 4.1.2 BLUF domain

**Figure 7:**
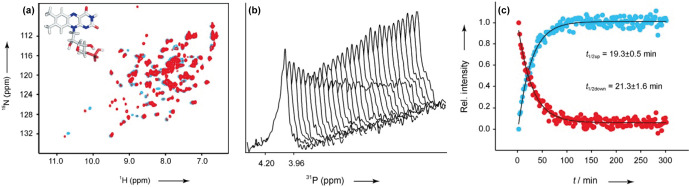
**(a)** ${}^{1}$ H, ${}^{15}$ N HSQC of YcgF (reconstituted with FMN) in the dark (blue) and light (red) states. **(b)** ${}^{31}$ P stack plot representations of the chromophore after light illumination. **(c)** Normalised and fitted signal intensities of ${}^{31}$ P kinetics traces for t1/2 calculations. Reproduced with permission from Wiley (Schroeder et al., 2008).

Proteins containing a BLUF (sensors of **b**lue-**l**ight **u**sing **f**lavin adenine dinucleotide) domain are another representative of proteins reacting to light. The BLUF domain is a flavin adenine dinucleotide (FAD)-binding domain and was found in various proteins, mainly present in proteobacteria, cyanobacteria and a few eukaryotic organisms (Gomelsky and Klug, 2002). In comparison to PYP, the chromophore in BLUF domains is not covalently bound (Wu and Gardner, 2009). The proteins detect blue light using their chromophore, followed by a reversible red shift and formation of a photo-activated conformation, the signalling state that decays spontaneously to the ground state if the system returns to a dark environment (Zirak et al., 2006). Illumination induces different structural and functional outputs as regulation of catalytic activity of enzymes and second messengers, photophobic responses and expression control of photosynthetic genes (Gomelsky and Klug, 2002). Besides other methods as time-resolved fluorescence or absorption spectroscopy, NMR coupled with light is a powerful tool for the investigation of the photoreaction mechanisms in BLUF domains after light irradiation at atomic resolution. The best-characterised BLUF photoreactions analysed by NMR are the ones of AppA (Gauden et al., 2007; Grinstead et al., 2006a, b), BlrB (Jung et al., 2005; Wu et al., 2008) (from *Rhodobacter sphaeroides*), BlrP1 (from *Klebsiella pneumonia*) (Wu and Gardner, 2009) and YcgF (from *Escherichia coli*) (Schroeder et al., 2008). Kaptein and his co-workers studied the AppA BLUF domain and presented a solution structure as well as evidence of structural changes in the light-induced state, e.g. for surface residues and the flipping of a glutamine side chain followed by the formation of a hydrogen bond (Grinstead et al., 2006b, a). By mutation of aromatic amino acids that are a short distance from the FAD cofactor, they were able to gain more information about the electron-transfer pathways in BLUF domains (Gauden et al., 2007). With NMR under light and dark conditions, Gardner and co-workers (Wu et al., 2008) observed structural changes in BlrB for amino acids near the flavin-binding pocket but also more than 1.5 nm apart. This finding indicates that the light-induced signal is propagated from the flavin through the protein, resulting in the initiation of the regulatory function (Wu et al., 2008). Together with the Essen group in Marburg, Schwalbe and coworkers investigated YcgF from *E. coli* (reconstituted with FMN and FAD) and, in comparison to HSQC spectra of AppA and BlrB, much stronger chemical shift perturbations were observed upon light excitation (Fig. 7a) (Schroeder et al., 2008). Furthermore, they recorded kinetics for the dark-state recovery of YcgF by proton NMR spectroscopy in a temperature-dependent manner. Additionally, ${}^{31}$ P kinetic measurements were performed to investigate the kinetic behaviour of the chromophore signals upon illumination (Fig. 7b–c). The results combined with UV/Vis spectroscopy show a heterogeneous distribution of half-life times for the light-to-dark conversion, suggesting hysteresis effects.

#### 4.1.3 Time-resolved NMR studies of the photocycle of visual rhodopsins by liquid-state and solid-state NMR

The characterisation of liquid- and solid-state NMR experiments of the dark state and various light states of rhodopsins has long been fascinating. Cryotrapping experiments on bacteriorhodopsin coupled to DNP have been pioneered in the groups of Griffin and Herzfeld (Mak-Jurkauskas et al., 2008; Ni et al., 2018). From a technical point of view, the NMR-spectroscopic time-resolved investigation of the photocycle of the eukaryotic, visual bovine rhodopsin, the mammalian visual dim-light G-protein-coupled photoreceptor, represents one of the most challenging biophysical studies for characterising key intermediates and kinetics of its photocycle as its photocycle is irreversible, highly light-sensitive and the spectra of functional rhodopsin of extremely low signal-to-noise. Such functional rhodopsin can only be prepared using eukaryotic expression systems (Reeves et al., 2002).

**Figure 8:**
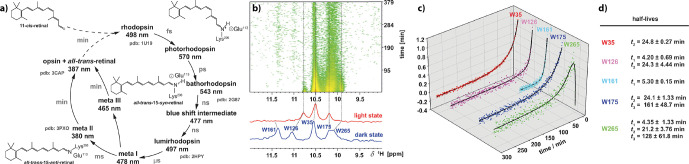
Analyses of the long-lived meta III and meta II states of rhodopsin after illumination. **(a)** The photocycle of bovine rhodopsin with the first detectable intermediate of photorhodopsin and subsequent intermediates as a product of thermal relaxation. **(b)** Time series of ${}^{1}$ H-NMR spectra from the indole region of a selective α , ε - ${}^{15}$ N-tryptophan-labelled rhodopsin and corresponding dark state (blue) and light state (red). **(c)** Time traces of the indole signal intensities and the corresponding half-lives extracted from exponential fits to the time-dependent signal intensities. The figure was adapted with permission from Wiley (Stehle et al., 2014).

Opsin, the apo-protein, is covalently attached to the chromophore 11-cis-retinal through Schiff base formation. Upon photon absorption, the retinal undergoes a E/Z isomerisation of the cis-configured double bond to all-trans-retinal. Thus, isomerisation induces conformational transitions and population of several high-energy photocycle intermediates, whose decay leads to the formation of the meta II signal state. The aim of our time-resolved studies was the characterisation of the decay kinetics of this meta-II state resolved on individual amino acid reporter signals. For this, we could assign the ${}^{1}$ H, ${}^{15}$ N tryptophan side-chain indole resonances using selectively ${}^{15}$ N-labelled tryptophan expressed in HEK293 cells (Werner et al., 2008), and we also pursued the attachment of fluorinated reported groups to cysteine thiol groups to utilise ${}^{19}$ F-NMR to follow these conformational changes in a time-resolved manner (Loewen et al., 2001). After in situ illumination of the dark state of rhodopsin, we could detect the NMR signals in the meta-II state in 2D correlation spectra and could analyse the decay kinetics of meta-II. These kinetics are bifurcated: next to the known formation of opsin, we could show the meta-III state to be populated (Fig. 8). This meta-III state is not signalling but considered a storage system (Stehle et al., 2014).

This finding is important, as continued activation of the photocycle of rhodopsin leads to the accumulation of all-trans-retinal in the rod outer segments (ROS). For retinal homeostasis, deactivation processes are required to delay the release of retinal. Bovine visual arrestin (Arr(Tr)) has been previously proposed to play a key role in the deactivation process, and in fact, time-resolved NMR together with optical spectroscopy conducted by the group of Wachtveitl could show that formation of the rhodopsin–arrestin complex markedly influences partitioning in the decay kinetics of rhodopsin. Binding of Arr(Tr) leads to an increase in the population of the meta III state that is simultaneously formed with meta II from meta I (Chatterjee et al., 2015). We further studied the retinal-disease-relevant G90D bovine rhodopsin mutant by time-resolved liquid-state and DNP-enhanced solid-state NMR with the group of Glaubitz as well as by advanced optical spectroscopy with the group of Wachtveitl (Kubatova et al., 2020). The G90D mutation is one of numerous mutations that impair the visual cycle of the mammalian dim-light photoreceptor rhodopsin; it is a constitutively active mutant form that causes congenital stationary night blindness (CSNB) disease. Different to previous crystallographic reports, we could detect two long-lived dark states, both of which contain the retinal in 11-cis configuration. By studying the photocycle with DNP-enhanced solid-state NMR, we could detect the dark state, the bathorhodopsin and the meta-II state and could show that all these states retain their conformational heterogeneity. This conformational heterogeneity is linked to a substantially altered photocycle as shown by optical spectroscopy.

**Figure 9:**
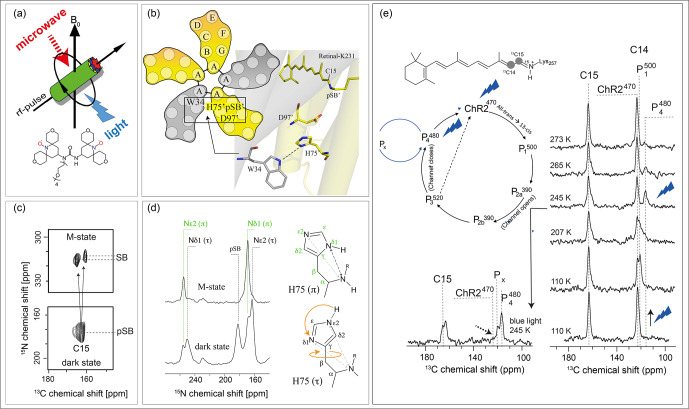
Example of cryogenic trapping of light-induced photoreceptor intermediates and detection by DNP-enhanced solid-state NMR. **(a)** An experimental setup is required which allows simultaneous light and microwave irradiation under MAS-NMR conditions at low temperatures. **(b)** The pentameric proton pump proteorhodopsin undergoes a photocycle with a number of distinct intermediate states, which can be trapped for DNP solid-sate NMR (Mehler et al., 2017). **(c)** Upon retinal isomerisation and Schiff base deprotonation, two distinct M states form as shown here by a NC-TEDOR spectrum. **(d)** In the M state, the tautomeric and rotameric state of H75, which forms a functionally important triad with proton acceptors D97 and W34 across the pentamer interfaces, changes (Maciejko et al., 2019). **(e)** The application of thermal trapping, relaxation and freeze-quenching protocols together with DNP enabled the first NMR analysis of the retinal chromophore within the light-gated ion channel channelrohodopsin-2 during the photocycle (Becker-Baldus et al., 2015).

DNP-enhanced solid-state NMR in combination with cryotrapping of light-induced intermediates of membrane-bound photoreceptors such as rhodopsins offers insight to link their 3D structures with their photochemical properties. Typical readout parameters are isotropic and anisotropic chemical shifts, homonuclear and heteronuclear dipole couplings or torsion angles by which the finest alterations within the chromophores during the photocycle could be detected (Becker-Baldus et al., 2015; Carravetta et al., 2004; Concistrè et al., 2008). The use of DNP in these systems was demonstrated for bacteriorhodopsin (Bajaj et al., 2009), and the discovery of many new rhodopsins inspired a series of new experiments covering the marine photoreceptor proteorhodopsin (Mehler et al., 2017), the light-gated ion channel rhodopsin-2 or the light-driven Na pump KR2 (Jakdetchai et al., 2021). In order to trap a desired photointermediate for DNP solid-state NMR analysis, an optimised MAS-NMR setup is needed, allowing simultaneous sample illumination by light with the desired wavelength as well as microwave irradiation (Fig. 9a). Furthermore, a suitable cryotrapping protocol has to be established, which depends on the particular photoreceptor properties and the targeted intermediate state (see Becker-Baldus and Glaubitz, 2018, for an overview) (Mak-Jurkauskas et al., 2008; Ni et al., 2018). An illustration of this approach is provided for the light-driven proton pump proteorhodopsin (Bamann et al., 2014) in Fig. 9b–d. Proteorhodopsin is the most abundant photoreceptor found today. Light-induced cryotrapping enabled the analysis of the M state, which is a key step for the proton transfer. This state forms after retinal isomerisation from all-*trans* to 13-*cis* and upon de-protonation of the Schiff base. The protein forms a homo-pentamer in the membrane with functionally relevant cross-protomer interactions (Fig. 9b). A DNP-enhanced TEDOR on the trapped M state reveals the formation of two distinct substrates (Fig. 9c) (Mehler et al., 2017). The residue H75, which links the proton acceptor D97 with W34 across the protomer interface, switches its tautomeric state and ring orientation during M-state proton transfer (Fig. 9d) (Maciejko et al., 2019). Another example is shown in Fig. 9e. Channelrhodopsin-2 is a light-gated ion channel. Its photocycle is linked to channel opening and closing by a still unknown mechanism. The chromophore retinal isomerises upon light absorption and the protein interconverts into various photocycle intermediate states. Most of these states could be trapped under DNP conditions by thermal trapping, relaxation and freeze-quenching protocols (Becker-Baldus et al., 2015).

### 4.2 Rapid mixing and photochemical triggering by photolabile protecting groups

#### 4.2.1 Proteins

A number of proteins have been investigated by time-resolved NMR using mainly rapid mixing, but also light induction of folding.

The small two-domain calcium-binding protein bovine α -lactalbumin (BLA) populates three different states depending on the buffer conditions applied: an unfolded state under denaturing conditions, a molten globule state at low pH (A state) and a native state under native conditions. The presence of Ca ${}^{2+}$ stabilises the protein and leads to increased folding rates in refolding experiments.

**Figure 10:**
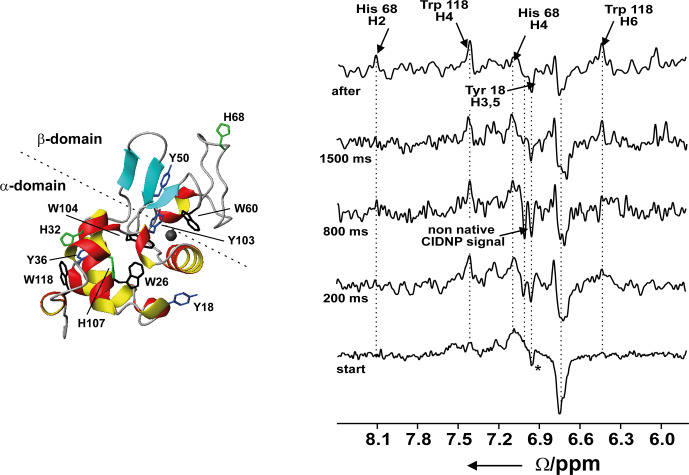
Time-resolved photo-CIDNP NMR of the refolding of bovine α -lactalbumin at 4M urea upon the addition of Ca2 + using laser irradiation. Left: ribbon representation of the native structure of the protein (1HFZ.pdb) (Pike et al., 1996); ${}^{*}$ is the native signal of Tyr18 which is already present at the start of the experiment. Reproduced with permission from Wiley (Wirmer et al., 2001).

We investigated the Ca ${}^{2+}$ -dependent transition from the unfolded to folded state of BLA in the presence of urea at neutral pH using photochemical triggering by releasing Ca ${}^{2+}$ from the photolabile chelator DM-Nitrophen (Kühn and Schwalbe, 2000; Schlepckow et al., 2008) using laser irradiation. We could show that folding under these conditions proceeds via parallel folding pathways (Schlepckow et al., 2008). Coupling of light-induced folding and photo-CIDNP using two lasers coupled into one quartz fibre allowed us to characterise a folding intermediate with a non-native environment that is populated already after 200 ms and disappears again after 1.5 s (Wirmer et al., 2001); see Fig. 10.

Balbach et al. (1995) showed as early as 1995 that a folding intermediate of refolding from the GdnCl-unfolded state of α -lactalbumin by rapid dilution resembles the molten globule of the protein by comparison of kinetic and static 1D spectra. In the second study (Balbach et al., 1996), they investigated the cooperative nature of folding from the molten globule state to the native state by raising the pH during mixing in the absence of denaturant. They extracted kinetic rates on a per-residue basis from one HSQC spectrum recorded during folding by simulating the observed line shapes. Schanda et al. (2007) directly measured folding rates of this folding process by implementing fluid turbulence-adapted SOFAST-HMQC measurements, which allowed them to record HMQC spectra every 10.9 s during the folding process (Schanda et al., 2007). They observed uniform mono-exponential folding rates throughout the molecule, confirming the presence of a single transition state.

Other proteins that were investigated using folding initiation by mixing and detection using 2D and 3D NMR spectroscopy include S54G/P55N ribonuclease $T_{1}$ (Haupt et al., 2011) and $\beta_{2}$ -microglobulin (B2M) (Franco et al., 2017b; Rennella et al., 2012). Beta-2-microglobulin (B2M) is an amlyloidogenic protein that folds via an intermediate which is presumably involved in the onset of aggregation. In two impressive studies, the group of Brutscher recorded 3D BEST-TROSY experiments during the course of folding in only 40 to 50 min and reconstructed the respective spectrum of the intermediate by comparing the real-time spectra with static spectra. Using this methodology, they were able to obtain 3D HNCA and 3D HNCO spectra as well as relaxation dispersion spectra of the intermediate. They could assign the intermediate and show that the intermediate is native-like. Furthermore, they found that the monomer–dimer transition is faster in the intermediate than in the native state.

The rapid-mixing technology used for the investigation of the proteins mentioned above can be combined with photo-CIDNP: refolding of hen egg white lysozyme (HEWL) (Dobson and Hore, 1998; Hore et al., 1997), the histidine-containing phosphocarrier protein HPr (Canet et al., 2003), and ribonuclease A (Day et al., 2009) have been studied.

**Figure 11:**
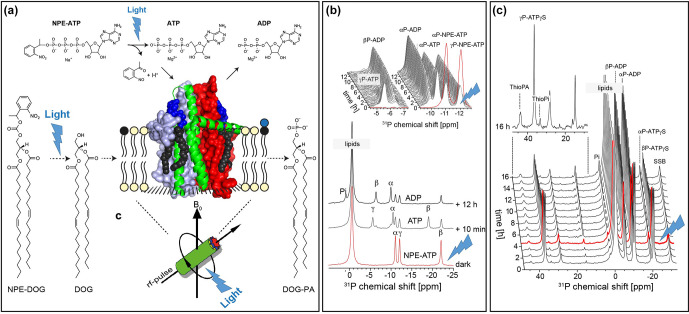
Proof-of-concept demonstration for photolytic uncaging of enzymatic substrates in proteoliposomes under MAS NMR conditions (de Mos et al., 2020): **(a)** *E. coli* diacylglycerolkinase (DGK) reconstituted into liposomes containing caged lipid substrate NPE-DOG or NPE-ATP. The proteoliposomes are illuminated by UV light in situ within the MAS rotor during the NMR experiment. **(b)** Successful uncaging of NPE-ATP followed by ATP consumption by DGK. **(c)** Uncaging of NPE-DOG results in the successful formation of Thio-PA under consumption of ATP- γ S.

The concept of photochemical triggering of biochemical processes by light is also appealing to solid-state NMR. So far, only a few examples have been reported in which reactions catalysed by membrane proteins have been followed by real-time MAS NMR spectroscopy (Kaur et al., 2016; Ullrich et al., 2011). Such studies are challenging since the nature of MAS-NMR makes the samples sealed within a MAS rotor inaccessible during the experiment, preventing titration of reagents. The reaction can therefore only be triggered for example by a T jump on a sample mixture stored in a pre-cooled MAS rotor. However, the feasibility of releasing substrates protected by photolabile groups directly during the MAS NMR experiment following in situ illumination has been recently demonstrated for the first time (de Mos et al., 2020): the *E. coli* lipid regulator diacylglycerolkinase phosphorylates its lipid substrate diacylglycerol under ATP consumption. It was possible to demonstrate that both ATP as well as the lipid substrate protected by a nitrophenylethyl group (NPE group) can be uncaged directly under MAS-NMR, triggering DGK's enzymatic activity, which could be followed by ${}^{31}$ P detection (Fig. 11).

While the previous examples of protein folding use cofactors or internal chromophores for the initiation of folding, site-specific photoprotection of amino acids has not been used in time-resolved NMR, yet. A breakthrough for the site-specific labelling of proteins for NMR was the incorporation of unnatural amino acids in vivo. By using an orthogonal tRNA/aminoacyl-tRNA synthetase pair, unnatural amino acids can be integrated into proteins in response to a TAG amber frame shift codon (Xie and Schultz, 2006). These site-specific incorporated unnatural amino acids are attractive for the investigation of ligand binding or protein folding in vitro and in vivo (Jones et al., 2009). Especially for NMR, isotope-labelled (19F, 13C, 15N; Cellitti et al., 2008; Hammill et al., 2007; Jackson et al., 2007) photo-caged, spin-labelled and metal-chelating (Lee et al., 2009; Otting, 2008; Xie et al., 2007) unnatural amino acids are of high interest. Here, we will focus on the photo-caged amino acids, represented e.g. by o-nitrobenzyl (o-NB) caged tyrosine (Deiters et al., 2006), cysteine (Wu et al., 2004), lysine (Chen et al., 2009), the 4,5-dimethoxy-2-nitrobenzyl caged serine (DMNB) (Lemke et al., 2007) and 1-Bromo-1-[4 ${}^{\prime }$ ,5 ${}^{\prime }$ -(methylenedioxy)-2 ${}^{\prime }$ -nitrophenyl]ethane caged selenocysteine (Welegedara et al., 2018). These caging groups have different photo-physical properties: o-NB and the selenocysteine are cleaved by UV illumination and DMNB by blue visible light. By inserting such a mutation into a protein, the function and structure can be modified and controlled. Cellitti et al. (2008) incorporated a photo-caged tyrosine into the active site of the 33 kDa thioesterase domain of human fatty acid synthase (FAS-TE) and could show inhibition of binding of the tool compound. After cleavage of the photo cage via UV light binding was reestablished, demonstrating that site-specific labelling via photo cages can be achieved without modifying the protein sequence but with the possibility of inhibiting and regenerating the function of the natural amino acid after cleavage. Another example of inactivation of function is the use of an o-NB caged cysteine at the active site of a pro-apoptotic cysteine protease caspase-3. After cleavage the natural amino acid is obtained, and 40 % of its activity is restored (Wu et al., 2004). Photo-caged amino acids can also be used to allow for selective covalent modifications in proteins after cleavage of the cage. With site-specific incorporation of a photo-caged selenocysteine and following uncaging, it is possible to site-specifically modify these due to their higher reactivity in comparison to competing cysteine residues (Welegedara et al., 2018).

#### 4.2.2 RNA

In time-resolved NMR studies characterising the folding or refolding of RNAs, two main strategies for the utilisation of photo-caged compounds can be employed. Either the RNA itself or a folding-inducing ligand can be modified (Fürtig et al., 2007b). Folding-inducing ligands include high-affinity, low-molecular-weight ligands, e.g. in riboswitch folding, or divalent ions, in particular Mg ${}^{2+}$ . If the RNA itself is modified, several strategies can be applied with regard to choice and positioning of the photolabile functional group. One approach is to modify the nucleobases in order to sterically and chemically prevent the formation of mutually exclusive base pairs within the different conformations whose interconversion shall be studied (Höbartner et al., 2004). The second approach is to place the photo cage at a functional group within the backbone of the RNA. This can be for example the 2'-OH group that is important in the establishment of stabilising interactions as well as being the mediator of RNA-catalysed reactions (Manoharan et al., 2009). For the minimal hammerhead ribozyme, caging of the active 2'OH- group at the active site in combination with position-selective ${}^{13}$ C labelling revealed a concerted motion of both nucleotides of the catalytic centre during the catalysed cleavage reaction (Fürtig et al., 2012, 2008).

**Figure 12:**
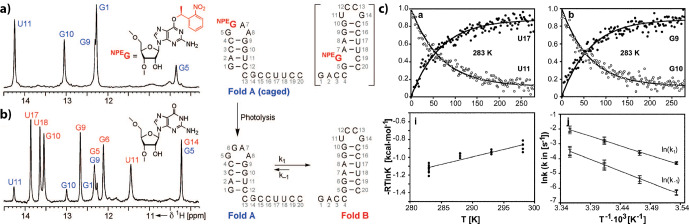
**(a, b)** ${}^{1}$ H NMR spectra of imino protons with colour coding according to the secondary structure shown to the right. **(a)** Spectrum of the conformationally locked Fold A (caged by introduction of O ${}^{6}$ -(S)-NPE modified guanosine at position 6). **(c)** Top: representative normalised signal intensities as a function of time after release of the native state by a single laser pulse. Down: Arrhenius analyses of $k_{1}$ and $k_{-1}$ . Reproduced with permission from Wiley (Wenter et al., 2005).

Application of the approach to cage the nucleobase led to the characterisation of refolding events in various bistable RNAs (Fig. 12) (Wenter et al., 2006, 2005) and to the formulation of generalised folding rules that correlate the number of base pairs with refolding rates (Fürtig et al., 2007a) and with the delineation of transition state conformations in RNA-refolding reactions (Fürtig et al., 2020, 2010). When applied to RNAs for which refolding is intimately linked to biological function, the exact folding pathways during transcription involving the metastable states could be determined (Helmling et al., 2018). The introduction of photo-caged protecting groups normally requires the production of the RNA by chemical solid-phase synthesis (Brieke et al., 2012; Mayer and Heckel, 2006), rendering the simultaneous incorporation of isotope-labelled nucleotides laborious and expensive (Quant et al., 1994). However, new chemo-enzymatic techniques that are able to combine chemically modified and in vitro transcribed, isotope-labelled strands within a single RNA resolve these difficulties (Keyhani et al., 2018) and will enable light-triggered folding studies of more sizeable and complex RNAs in the future. Likewise, tremendous advances have also been made in the chemistry of photo-protecting groups utilised to cage RNAs. Whereas early studies mainly focus on the 1-(2-nitrophenyl)ethyl that needs to be cleaved with UV light and has a limited steric demand (Ellis-Davies and Kaplan, 1988), new concepts using photo-caging groups with either more red-shifted absorption or higher destabilising potency have emerged (Ruble et al., 2015; Seyfried et al., 2018). Right from the beginning, the methodology of caging interacting ligands could be utilised in the study of more complex RNA systems at the size limit of liquid-state NMR. The first studies on the aptamer domain of the guanine-sensing riboswitch where the ligand hypoxanthine was caged revealed a two-state folding trajectory (Buck et al., 2007). This is an important molecular feature that enables fast discrimination of cognate over near-cognate ligands and enables the kinetic control of transcription termination within the two-domain full-length riboswitch (Steinert et al., 2017). In this application, resolving the folding dynamics at the level of individual nucleotides was only possible by application of nucleotide-type selective isotope labelling in conjunction with x-filtered and X-nuclei-edited real-time NMR experiments.

Caging of divalent ions is challenging for RNA as they are often needed for proper folding of tertiary interactions, but also as the affinities stay in the micromolar to millimolar range. However, for the Diels–Alder ribozyme the difference in reactivity for different mutants could be traced down to the differences in local dynamics around the catalytic pocket (Manoharan et al., 2009). In this case, besides mixing, release of the divalent ions from a photo-caged chelator was also possible (Fürtig et al., 2007b).

More recently we also investigated folding of RNA using the rapid-mixing methodology. Here, folding can be induced e.g. by rapid mixing of metal ions, as has been exemplified for ribozymes by addition of Ca ${}^{2+}$ (Manoharan et al., 2009) and structural changes in RNA riboswitches by adding their specific ligand (Reining et al., 2013). All these applications share the detection of the kinetics on the imino-resonances in 1D spectra.

#### 4.2.3 DNA and RNA non-canonical structures – time-resolved NMR studies of DNA and RNA G-quadruplexes and DNA i-motif folding and refolding

Non-canonical DNA structures including G-quadruplexes (G4) and i-motifs typically coexist in several heterogeneously folded conformations. This pronounced structural polymorphism and the associated inherent dynamic character make these structural motifs prime examples of time-resolved NMR studies. The pH-induced folding of a DNA i-motif revealed that the folding follows kinetic partitioning, with re-equilibration processes subsequent to the initial folding (Lannes et al., 2015; Lieblein et al., 2012). Similar findings have been made for a telomeric DNA G4 that coexists in two conformations with different folding topologies. For G4 DNA, folding can be induced by rapid injection of a K ${}^{+}$ -buffer solution, since monovalent cations are essential for G4 formation. DNA G4 folding follows complex folding pathways, which involve long-lived intermediate states that persist for several hours, and the re-equilibration proceeds over days at room temperature (Bessi et al., 2015). The folding energy landscapes for RNA G4s are however markedly different compared to DNA G4s, with significantly faster and monophasic folding kinetics (Müller et al., 2021).

**Figure 13:**
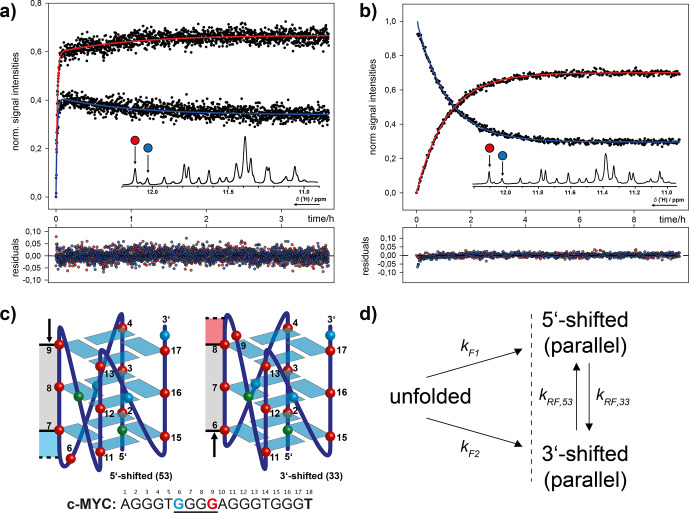
Combinatorial study of folding and refolding of a 18-mer DNA G-quadruplex (G4)-forming oligonucleotide sequence from the human cMYC proto-oncogene promoter by Grün et al. (2020). **(a)** Parallel folding of two coexistent G4 conformations, induced by in situ rapid mixing with K ${}^{+}$ ions that are required for G4 formation. **(b)** Refolding of photo-caged, isolated G4 conformations back into conformational equilibrium. **(c)** Schematic representation of the two coexisting G4 conformations distinguished by a register-shifted G-rich strand. **(d)** Kinetic model for parallel folding into two coexisting conformations and subsequent refolding dynamics in conformational equilibrium. Reprinted (adapted) with permission from *Journal Of The American Chemical Society* 2020 (Grün et al., 2020). © 2020 American Chemical Society.

Recently, we investigated the folding and refolding kinetics of an 18-mer DNA (G4)-forming oligonucleotide sequence from the human *cMYC* proto-oncogene promoter (Fig. 13). This G4 coexists in two conformations that are distinguished by a register shift of one G-rich strand segment along the stacked tetrads. To study the conformational dynamics in this system, we used a combination of K ${}^{+}$ -induced folding with an approach where we photochemically trapped a single conformation and induced refolding with in situ laser irradiation. By site-specific incorporation of photo cages, we could block the base pair interactions for distinct nucleotides. This strategy allowed us to separate the two conformations and study the refolding mechanism in detail. The proposed kinetic model, based on kinetic and thermodynamic experimental data, reveals that after initial folding the two conformations can directly refold into each other. The proposed transition state requires only a minimal degree of unfolding. The slow refolding kinetics (0.9 h ${}^{-1}$ ) are caused by a relatively high activation energy that is needed for an initial opening of the base paired tetrads. Further, we showed that folding kinetics induced by rapid mixing deviate by several orders of magnitude from light-induced folding. This finding highlights that the altered energy landscapes under different non-equilibrium conditions have a severe impact on the folding dynamics. Photolabile protecting groups here are an optimal tool for investigating native, unmodified (after photocleavage) oligonucleotide dynamics under constant experimental (pressure, temperature, buffer composition) and physiological conditions (Grün et al., 2020). More recently, we have disentangled the complex folding energy landscape of the *cMYC* G4 that involves conformational dynamics of different G-strand isomers (*spare-tire* isomers). Here, the photo-caging strategy was used to trap completely unfolded states, which yields unbiased folding kinetics (Grün et al., 2021).

### 4.3 Overview of non-light applications

#### 4.3.1 Temperature jump

There have been several different techniques for T -jump experiments in combination with real-time NMR spectroscopy, but so far applications on biomolecular systems have been limited. In most cases, RNase A was used as a test system to either follow its heat-denaturation kinetics (Akasaka et al., 1991; Kawakami and Akasaka, 1998) or, in the case of a flow system (Yamasaki et al., 2013), the refolding from its heat-denatured state was followed. In all cases simple two-state kinetics were observed.

**Figure 14:**
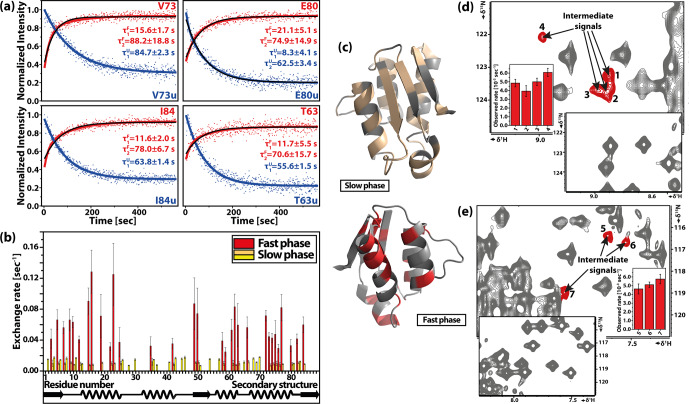
T -jump experiment on the cold-denatured C40A/C82A barstar mutant. **(a)** Representative time-dependent signal intensities with double-exponential kinetic fit. **(b)** Residue-specific results of refolding kinetics. **(c)** Three-dimensional structure of barstar (PDB: 1AB7) highlighting the affected residues with slow (pale yellow) and fast (red) phases of observed kinetics. **(d, e)** Different part of the same ${}^{1}$ H– ${}^{15}$ N HSQC spectrum detected in the slow T -jump experiment using a gas-heating system with high-sensitivity cryoprobe-detected stable intermediate signals. Reproduced with permission from Pintér and Schwalbe (2020).

The most recent application of rf heating in combination with cold-denatured barstar allowed detailed characterisation of different folding pathways. A stable intermediate was observed on the slow folding pathway (Fig. 14), where the rate limiting step is the *trans-cis* isomerisation of the Tyr47-Pro48 amide bond. Additionally, the reversibility of the system and the slow *cis-trans* isomerisation allowed the measurement of double-jump experiment to study an alternative folding pathway. The equilibrium-folded barstar was denatured by a short cooling (2.5 min) time, keeping the Tyr47-Pro48 residue in *cis* conformation. From this non-equilibrium denatured state, a state-correlated spectrum was used. Although reaction rates could not be determined, evidence for an ensemble of intermediates was observed.

#### 4.3.2 Pressure jump

Slow kinetics induced by pressure jump can be utilised to follow the pressure-induced dissociation or re-association of amyloid fibrils (Kamatari et al., 2005; Niraula et al., 2004) or prion fibrils (Akasaka et al., 2014) providing detailed information about the formation of different intermediate states. Additionally, thanks to the technical advancements in the last 10 years, not only static, but also pressure-jump experiments can be utilised in combination with RT-NMR spectroscopy to study protein folding and kinetics.

First, Kremer et al. (2011) showed in 2011 that a pressure jump of around 800 bar can be achieved in both directions to de- or re-nature the protein. They used a model protein histidine-containing phosphocarrierprotein (HPr) to design and demonstrate new pulse sequences to study protein folding. The pressure change is an order of magnitude faster than the longitudinal relaxation time of proteins; therefore, it can be directly incorporated into the pulse sequence. It is a similar approach to the SCOTCH experiment designed by Rubinstenn et al. (1999) for light-sensitive proteins. Their approach was either at the start of the pulse sequence (pressure perturbation transient state spectroscopy – PPTSS) or during the pulse sequence (pressure perturbation state correlation spectroscopy – PPSCS) to change the pressure. With these experiments they could demonstrate how pressure can be introduced as a new dimension into the pulse sequence and measure the $k_{\mathrm{UN}}$ and $k_{\mathrm{NU}}$ of HPr.

Roche et al. (2013) focused on the staphylococcal nuclease (SNase) with a relatively long relaxation time to the new equilibrium state (even up to 24 h), allowing even manual pressure perturbation (Roche et al., 2013). They investigated the effect of the introduction of different cavities by mutations and their effect on the folding kinetics. They observed drastic changes in terms of both stability and folding pathways. The I92A mutant showed a structurally heterogeneous ensemble at the folding barrier with multiple folding pathways, while the WT SNase and hyperstable D+PHS mutants have a well-defined transient state and folding pathway.

The next major developments come from Charlier et al. (2018c), Alderson et al. (2017) and Charlier et al. (2018b, a) in a series of publications. Their new P -jump system as discussed in Sect. 2.3 allows, in both directions, very fast pressure change. Their pulse sequence approaches are similar to what Kremer et al. (2011) have shown already, but they developed even more complex ways of incorporation into the pulse sequence and of study of the folding mechanism of the pressure-sensitive ubiquitin. First, the combination of H/D exchange with pressure jump revealed biexponential refolding kinetics attributed to an off-pathway oligomeric intermediate. Next, the use of P jump in combination with 3D NMR spectroscopy allowed the chemical shift and ${}^{15}$ N transverse relaxation analyses of the still unfolded protein but already at low pressure. This revealed a very short-lived intermediate, which is different from the one revealed by hydrogen exchange. They proposed a folding mechanism of ubiquitin where two parallel but similarly efficient folding pathways take place: direct folding with no intermediate and folding via a short-lived intermediate state. Finally, they could prove by incorporating double-pressure jump into the pulse sequence and measuring the chemical shifts that this short-lived intermediate closely resembles the folded state with differences as they write in “the C-terminal strand, β 5, and its preceding loop, strand β 1, and the C-terminal residues of strand β 3, with β 5 being sandwiched between β 1 and β 3 in the natively folded state”.

#### 4.3.3 “Slow” kinetics

A number of processes are slow enough to be investigated by time-resolved NMR without the need for any device to initiate folding. One example is the formation of very large macromolecular assemblies. The group of Boisbouvier studied for example the self-assembly pathway of the 0.5 MDa proteolytic machinery TET2 (Macek et al., 2017), the group of Schwarzer investigated histone modification by time-resolved NMR spectroscopy (Liokatis et al., 2016).

The investigation of protein modification and in particular phosphorylation was put forward by Selenko in 2010 and has been used since then in a couple of applications (Kosten et al., 2014, p. 129; Landrieu et al., 2006; Liokatis et al., 2010; Mylona et al., 2016). Such studies are even possible in cellular environment by time-resolved in-cell NMR (Theillet et al., 2013). Other applications of time-resolved in-cell NMR are the proteolytic alpha-synuclein processing (Limatola et al., 2018), the methylation of lysines in cells, the investigation of ligand binding in cellular environments (Luchinat et al., 2020b, a) and the modulation of bound GTP levels of RAS (Zhao et al., 2020). Time-resolved NMR experiments are now also pursued to characterise metabolic flux in patient-derived primary cells (Alshamleh et al., 2020; Reed et al., 2019).

The formation of amyloids is another highly relevant slow process. While fast-tumbling monomers and flexible tails are amenable to liquid-state NMR spectroscopy, residues in intermediate exchange present in oligomers or residues in fibrils cannot be observed. Therefore, the aggregation is monitored as monomer loss kinetics following the signal decrease in the resonances present at the beginning of the experiment.

The misfolding of the prion protein into fibrils is observed in neurogenerative prion diseases. While the prion protein is a mainly α -helical globular protein with an unfolded tail in its native state, β -sheet structures are enriched in the polymeric fibrillar forms. We investigated the kinetics of fibril formation from the unfolded state on a per-residue basis of human prion protein (Kumar et al., 2010) and murine prion protein (Schlepckow and Schwalbe, 2013). Comparison of HSQC spectra directly dissolving the human protein (90–230) and after 4 and 7 d reveals that signals are lost quickly for the core of the fibril (145–223), while N-terminal signals decrease more slowly and signals close to the C-terminal (224–230) change their chemical shift, indicating a structural change in the latter region within the fibril. A more detailed view of the fibril formation was obtained on the murine prion protein, where signal loss states could be obtained from multiple HSQC measurements on a per-residue basis. Here we found that residues in close proximity to the disulfide bridge (C179–C214) broaden first, which we attribute to initial molecular contacts in oligomer formation, while in a second stage of the aggregation fibrils are formed.

**Figure 15:**
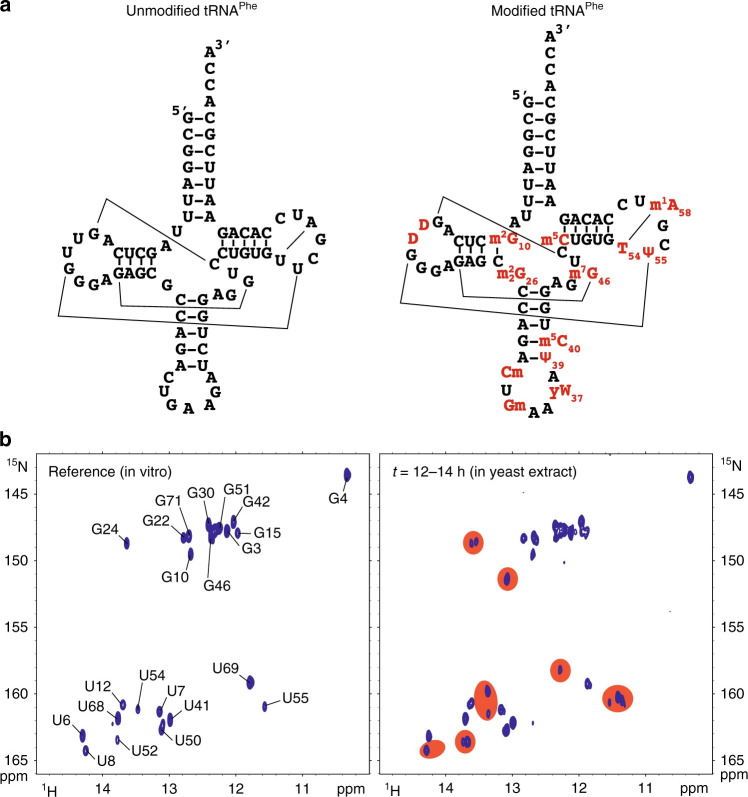
An RNA secondary structure of unmodified and modified tRNA ${}^{\mathrm{Phe}}$ a sequence and cloverleaf representation of unmodified yeast tRNA ${}^{\mathrm{Phe}}$ (left) and modified tRNA ${}^{\mathrm{Phe}}$ (right). **(b)** ${}^{1}$ H– ${}^{15}$ N HSQC spectra in vitro (left) and upon 12 h after addition to yeast extract. Assignments are indicated in the in vitro spectrum, while red patches highlight areas with significant changes. Reprinted from Barraud et al. (2019).

Another disease attributed to protein misfolding is Alzheimer's disease, in which A β peptides form fibrils. The aggregation of A β 1–40 and A β 1–42 has been investigated by a number of groups (Bellomo et al., 2018; Pauwels et al., 2012; Roche et al., 2016) by monitoring the loss of the monomers. Using the decay of the methylgroup region in a proton 1D as a reporter of aggregation, Luchinat and coworkers (Bellomo et al., 2018) conducted extensive parallel simulations on the folding kinetics at different initial monomer concentrations, applying a range of different models. Kinetics of amyloid formation can be best described by a model in which oligomers are formed which transform irreversibly into fibrils at a certain oligomer size. These fibrils grow (by addition and release of monomers) and undergo fibril fragmentation, resulting in smaller fibrils that in turn grow further.

Switching gears completely: another impressive example of the application of liquid NMR spectroscopy for the investigation of “slow” kinetics is the study of tRNA maturation. Barraud et al. (2019) were able to investigate the enzymatic modification of tRNA ${}^{\mathrm{Phe}}$ in yeast cell extract over 26 h using a series of HSQC spectra. Figure 15 shows HSQC spectra of the maturation after 12–14 h after addition of the tRNA ${}^{\mathrm{Phe}}$ to yeast extract, showing that modifications are inserted in a specific order along a defined route. This application nicely demonstrates the power of NMR to investigate complex mechanisms at the per-site resolution.

## 5 Conclusions

In this review, we have discussed the application of time-resolved NMR studies to study biomacromolecular folding, refolding, modification and aggregation. These studies utilise the power of NMR spectroscopy to determine kinetics of structural transitions together with site resolution. Different to other structural techniques, NMR not only provides snapshots of folding trajectories, but also provides positive evidence for the transition of two or several conformational states. It can determine the associated kinetic rates with rates as fast as 5000 s ${}^{-1}$ in a significant temperature range, allowing classification of structural transitions to follow Arrhenius or non-Arrhenius behaviour. Particularly interesting are biomolecular systems whose folding trajectory is subject to kinetic partitioning. Starting from a single state, folding pathways diverge and multiple folding pathways are populated, with kinetically or thermodynamically driven conformational states. Together with mutational studies, NMR is key in delineating transition-state characteristics and in detecting lowly populated states, and prime examples have been reported for proteins (Korzhnev et al., 2004) and for DNA (Kimsey et al., 2018) and their complexes (Afek et al., 2020). Future applications, thanks to unstoppable developments to increase signal-to-noise and resolution in NMR, will devise more sophisticated experiments to characterise transient conformations that often represent the key states carrying the biomolecular function.

## Data Availability

No data sets were used in this article.
